# Deep-agriNet: a lightweight attention-based encoder-decoder framework for crop identification using multispectral images

**DOI:** 10.3389/fpls.2023.1124939

**Published:** 2023-04-18

**Authors:** Yimin Hu, Ao Meng, Yanjun Wu, Le Zou, Zhou Jin, Taosheng Xu

**Affiliations:** ^1^ School of Big Data And Artificial Intelligence, Hefei University, Hefei, China; ^2^ Institute of Intelligent Machines, Hefei Institutes of Physical Science, Chinese Academy of Science, Hefei, China; ^3^ Science Island Branch, University of Science and Technology of China, Hefei, China

**Keywords:** multispectral image, crop identification, feature extraction, encoder-decoder, lightweight, DeepLab v3+

## Abstract

The field of computer vision has shown great potential for the identification of crops at large scales based on multispectral images. However, the challenge in designing crop identification networks lies in striking a balance between accuracy and a lightweight framework. Furthermore, there is a lack of accurate recognition methods for non-large-scale crops. In this paper, we propose an improved encoder-decoder framework based on DeepLab v3+ to accurately identify crops with different planting patterns. The network employs ShuffleNet v2 as the backbone to extract features at multiple levels. The decoder module integrates a convolutional block attention mechanism that combines both channel and spatial attention mechanisms to fuse attention features across the channel and spatial dimensions. We establish two datasets, DS1 and DS2, where DS1 is obtained from areas with large-scale crop planting, and DS2 is obtained from areas with scattered crop planting. On DS1, the improved network achieves a mean intersection over union (mIoU) of 0.972, overall accuracy (OA) of 0.981, and recall of 0.980, indicating a significant improvement of 7.0%, 5.0%, and 5.7%, respectively, compared to the original DeepLab v3+. On DS2, the improved network improves the mIoU, OA, and recall by 5.4%, 3.9%, and 4.4%, respectively. Notably, the number of parameters and giga floating-point operations (GFLOPs) required by the proposed Deep-agriNet is significantly smaller than that of DeepLab v3+ and other classic networks. Our findings demonstrate that Deep-agriNet performs better in identifying crops with different planting scales, and can serve as an effective tool for crop identification in various regions and countries.

## 1 Introduction

Timely identification of large-scale crops is vital for agricultural production, which can provide an important basis for yield estimation, structure adjustment and optimization of agricultural management (Becker-Reshef et al., 2010). The traditional identification methods of farm crops are mainly based on statistical statement, but the outdated method restricts identification efficiency and increases labor costs (Tan et al., 2020). Recently, a variety of automated detecting technologies have been proposed in crop identification and achieved lots of successful applications (Waldhoff et al., 2017; Longato et al., 2019; Xu et al., 2019). Remote sensing, as a large-scale non-contact monitoring technology, plays an extremely important role in modern agriculture (Shi et al., 2019). Identification of farm crops in remote sensing images in large-scale farmland can obtain the spatial location information of farmland and the ground attachment. The related information helps agricultural administrators to figure out the distribution and planting structure of regional species from a macro perspective, thereby formulate more accurate and efficient agricultural policies.

Crop identification based on remote sensing has been a research theme of considerable interest, which is of great value in the field of precision agriculture. With the development of image processing and artificial intelligence, the technologies of crop identification can be summarized into three streams. In the first stream, the traditional remote sensing feature extraction is mainly based on spectral, spatial, and temporal features (Zhang et al., 2016; Qiong et al., 2017; Sun et al., 2019b). Tian et al. (2021a) analyzed spectral characteristics and vegetation indices at each growth stage of crops and used reasonable thresholds to screen these parameters and successfully identified winter wheat and garlic planting areas. The result shows that varying vegetation indices could effectively distinguish crops with different spectral characteristics. Li et al. (2015) used the Stepwise Discriminant Analysis (SDA) method for feature selection from the Landsat MODIS Enhanced time series data and screened out 10 optimal features for crop classification. In the second stream, Machine learning methods are widely used in the field of large-scale crop identification due to their heuristic learning strategy and accelerated training mechanism (Jia et al., 2019; Zhang et al., 2019; Tian et al., 2021b). Zheng et al. (2015) applied Support Vector Machines (SVMs) to time-series Landsat images of Arizona to test its ability to discriminate between multiple crop types in a complex cropping system. Han et al. (2022) extracted relevant features of corn lodging regions and proposed the SMOTE-ENN-XGBoost model based on the Synthetic Minority Oversampling Technique (SMOTE) and Edited Nearest Neighbor (ENN) methods, which showed an F1 score of 0.930 and a recall rate of 0.899 on the lodging detection test set. With the proposal of the Convolutional Neural Network (CNN), deep learning leads the third stream for crop identification using remote-sensing images (Kuwata and Shibasaki, 2015; Wang et al., 2020; Yuan et al., 2020). Yu et al. (2022) improved the U-Net network by introducing the Involution operator and Dense block module and proposed a wheat lodging evaluation method based on UAV multispectral images. Kussul et al. (2017) proposed a multi-level Deep Learning (DL) method using multi-temporal land cover and crop type classification to identify crops in a heterogeneous environment, achieving a target accuracy of 85% for major crops. The proposal of attentional mechanisms has dramatically advanced the field of deep learning. Naturally, this ingenious mechanism has also been widely used for crop identification with great success (Jin et al., 2021). Wang et al. (2022) proposed a novel architecture called Coupled CNN and Transformer Network (CCTNet), which combines the local details of CNN and the global context of the transformer to achieve a 72.97% mIoU score on the Barley remote-sensing dataset. Lu et al. (2022) proposed a deep neural network with Dual Attention and Scale Fusion (DASFNet) to extract farmland from GF-2 images of southern Xinjian. The result shows that the dual attention mechanism module can correct the shape and boundary of the fields effectively.

The above methods show excellent performances in their respective datasets. However, these datasets are mainly derived from areas where crops are grown on a large scale. In fact, the vast majority of China’s regions are planted discretely, and the plots under this type of planting are relatively tiny, making it difficult for existing networks to achieve high-precision crop identification. In addition, a high-precision network is often accompanied by a considerable amount of parameter calculation, which is difficult to be applied to low-end agricultural equipment. Therefore, a highly accurate and lightweight neural network is urgently needed for agricultural production.

In this paper, we proposed a lightweight attention-based encoder-decoder framework for crop identification, and summarize the contributions of this paper as follows:

- Designed a lightweight network structure with much smaller parameters and floating-point of operations than DeepLab v3+ and other classical networks.
- Got an excellent identification accuracy, which can reach more than 98% accuracy in large-scale plots and more than 97% accuracy in small-scale plots.
- We also built two datasets corresponding to regular large-scale plots and irregular small-scale plots to test the performance of the Deep-agirNet in different environments.

## 2 Materials and methods

### 2.1 Study area

**Figure 1:**
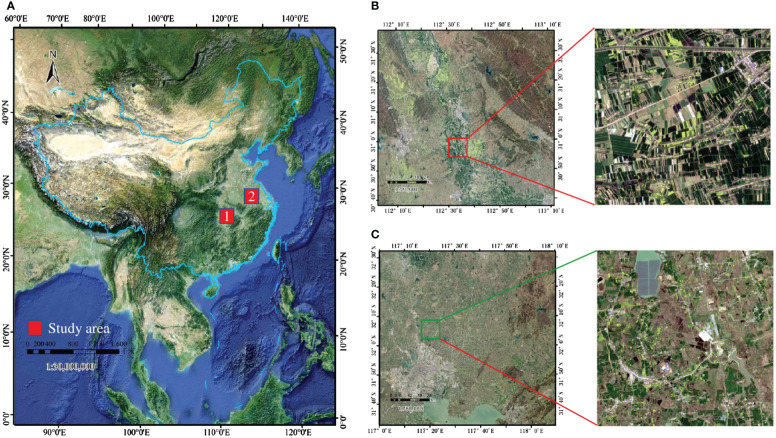
**(A)** Locations of the study areas in China (red), **(B)** Sentinel-2 image of the first study region, **(C)** Sentinel-2 image of the second study region.The red hollow rectangle shows the crop cultivation in the two regions.

As the most important winter crop in China, especially in the Yangtze River basin, winter wheat and canola have similar planting cycles, generally sown in September to October and harvested in April to May of the following year. Given that winter wheat and winter canola are important components of the agricultural economy, it is significant to know the distribution of these two crops for agricultural production and policy making. In this paper, two representative regions in the middle and lower reaches of the Yangtze River in China are selected as study areas, and their geographical locations are shown in **Figure 1**:

The first rectangular study area T49RFQ is bounded by longitudes 112°0*'* to 113°10*'* E and latitudes 30°40*'* to 31°40*'* N which mainly belongs to Jingmen City, Hubei Province. Jingmen City is planted on a large scale with regular and continuously distributed plots, which makes it easy to plant and harvest. The second rectangular study area, T50SNA, is bounded by longitudes 117°0*'* to 112°10*'* E and latitudes 31°30*'* to 32°30*'* N which mainly belongs to Hefei, Anhui Province. Hefei is planted discretely, with small and scattered plot sizes and low land use.

### 2.2 Remote sensing images processing

Sentinel 2 is a high-resolution multispectral imaging satellite built by the European Space Corporation and consisted of the “twin” satellites Sentinel 2A and Sentinel 2B. The remote sensing images taken by the Sentinel satellites contain 13 bands with different spatial resolutions (10m, 20m, 60m). In this study, all bands except Band 1 (Coastal aerosol), Band 9 (Water vapor), and Band 10 (SWIR-cirrus), which have the lowest spatial resolution, were screened and excluded, and a bilinear interpolation algorithm was applied to Band 5, 6, 7 (Red edge), Band 8b (Narrow NIR) and Band 11, 12 (SWIR) are resampled to a spatial resolution of 10m, and then these bands are fused to obtain a 10-channel remote sensing image with 10m spatial resolution.

**Figure 2:**
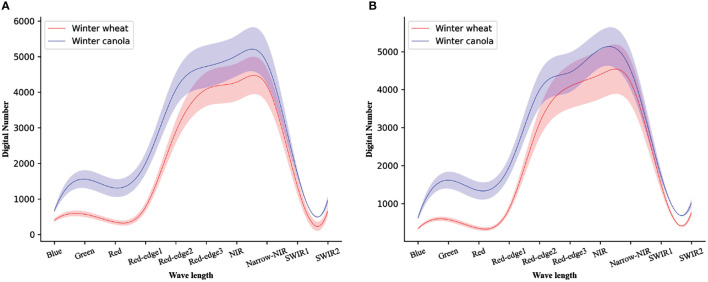
The Digital Number distribution plot of a multispectral of **(A)** the first study region of T49RFQ, **(B)** the second study region of T50SNA. Solid lines represent means and shaded areas represent one standard deviation from the mean.


Since crops of different planting scales will have different Digital Number (DN) distributions in remote sensing images, as shown in **Figure 2**. To reduce the errors caused by DN, percentage linear stretching is adopted in this study for each band of T50SNA:

(1) $$result=\frac{DN-min_{in}}{max_{in}-min_{in}}\times \left(max_{out}-min_{out}\right)+min_{out}$$

here *max_in_* and *min_in_* represent the maximum and minimum of DN of the stretched image, then *max_out_* and *min_out_* represent the set maximum and minimum of DN, respectively. Specifically, the maximum and minimum values of DN of T49RFQ are setas the upper and lower limits of pixel values. Then, the other image is linearly stretched to the set range so that the DN of the two images is finally distributed in the same range to reduce the deviation.

### 2.3 A lightweight encoder-decoder network based on DeepLab v3+

Since the first time used fully convolutional neural networks (Long et al., 2015) for end-to-end segmentation of natural images, semantic segmentation tasks for pixel-level classification have achieved leap-forward development. The vast majority of state-of-the-art (SOTA) segmentation networks, such as U-Net (Ronneberger et al., 2015), PSPNet (Zhao et al., 2017), DeepLab (Chen et al., 2017), and HRNetv2 (Sun et al., 2019a) are built based on encoder-decoder architecture. As an excellent semantic segmentation model with the encoder-decoder structure, DeepLab v3+ (Chen et al., 2018) is widely used in the field of semantic segmentation of remote sensing images. The part of the decoder includes Atrous Spatial Pyramid Pooling (ASPP) and an improved Xception module, where the ASPP module can control the size of the perceptual field by adjusting the expansion coefficient to capture the features at different scales. Then, two quadruple upsampling are used in the decoder part, where the first upsampling concatenates the low-dimensional features from the decoder and encoder to make features fusing, and the second upsampling restores the concatenated result to the same scale of inputs and classifies each pixel to obtain the segmentation result finally.

Despite the excellent performance of DeepLab v3+, it is hard to accept for agricultural production due to the large parameters. Considering a network serving agricultural production must balance accuracy and parameters, we made a lightweight improvement to DeepLab v3+ and named the improved network as Deep-agriNet.

**Figure 3:**
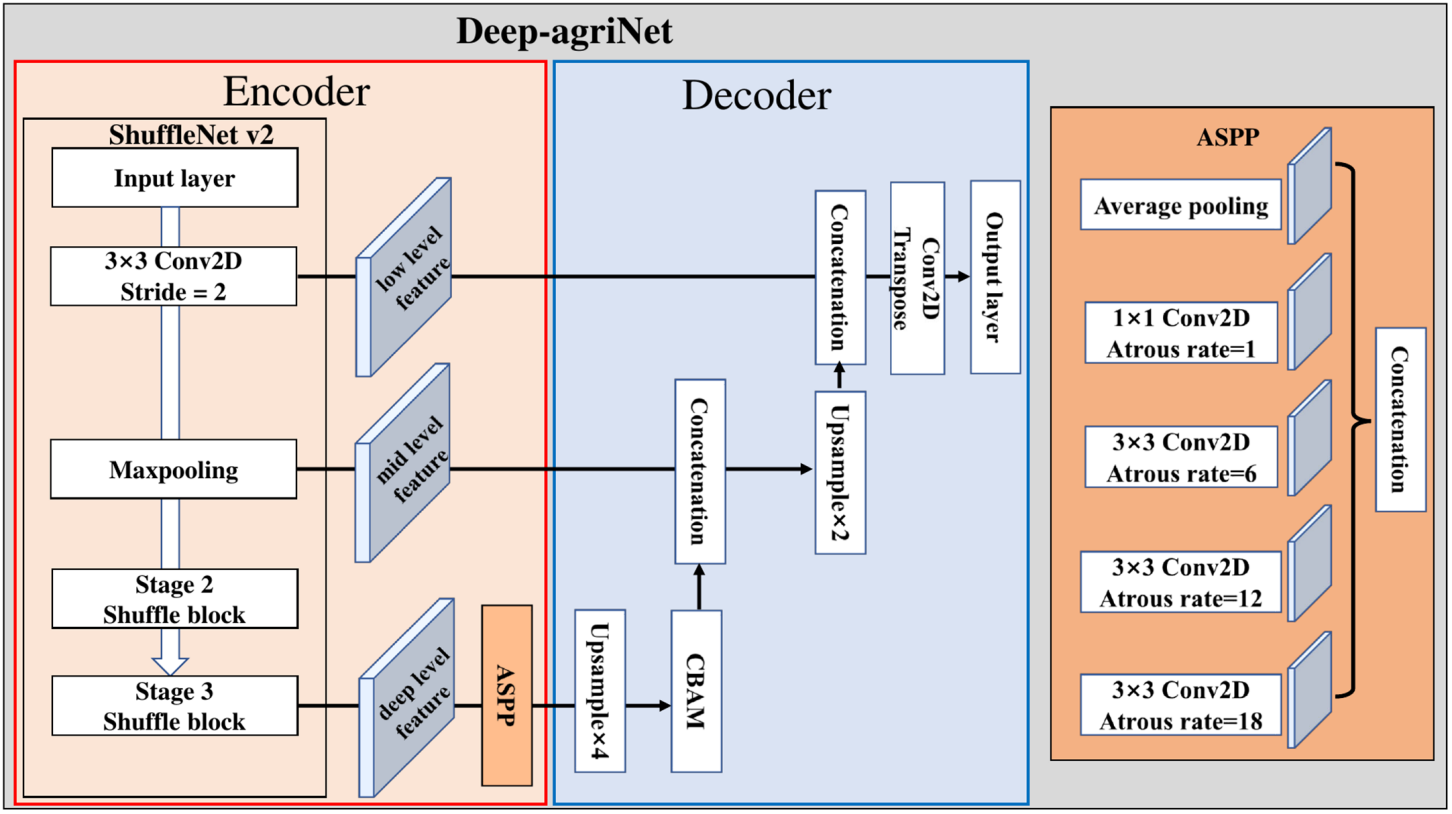
The framework of the Deep-agriNet.

As shown in **Figure 3**, the improvement of the network is mainly reflected in the following parts: In the encoder part, we chose ShuffleNet v2 (Ma et al., 2018), an advanced lightweight network architecture, as the feature extractor of Deep-agriNet. The design of ShuffleNet v2 is based on four network design criteria:

- Keeping the numbers of input and output channels equal minimizes memory access cost.
- A large group number used in group convolution increases computational cost.
- Complex network structure (abuse of branches and basic units) reduces the degree of network parallelism.
- The costs of element-wise operations cannot be neglected either.

**Figure 4:**
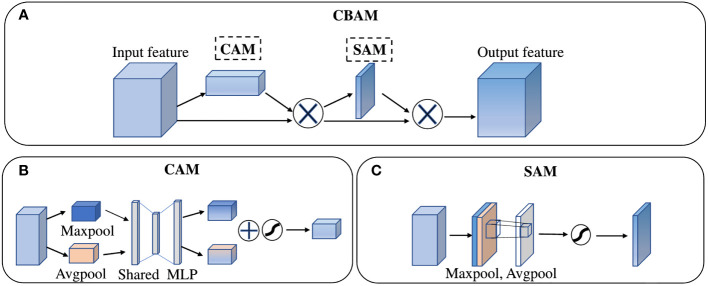
The structure of **(A)** Convolutional Block Attention Module, **(B)** Channel Attention Module, **(C)** Spatial Attention Module.

The operation of channel split was used in the basic shuffle unit of ShuffleNet v2. Then, it divided input channels evenly into two branches to replace the group convolution. As shown in **Figure 4A**, one branch of the basic shuffle unit does nothing to reduce network computation, and the other branch maintains the same number of channels in each convolution. The shuffle unit for spatial down sampling, as shown in **Figure 4B**, removes the channel split and doubles the number of output channels compared to the input channels. In addition, the outputs of both shuffle unit are no longer an add operation between elements but a concatenation, which can fuse the extracted features or output information instead of simple superposition. Finally, the results of concatenation are shuffled at the end of the basic unit by the channel shuffle operation to increase the information exchange between channels, thus improving the network performance.

**Figure 5:**
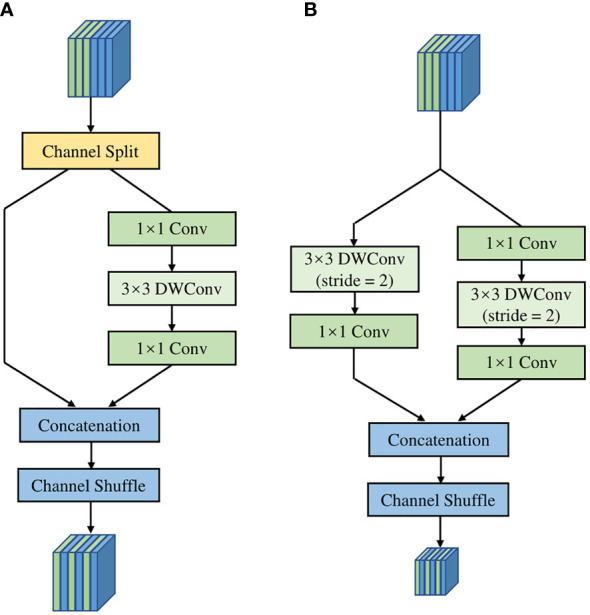
The structure of **(A)** basic unit, **(B)** unit for spatial down sampling.

In the decoder part, a Convolutional Block Attention Module (CBAM) (Woo et al., 2018) was added to the DeepLab v3+ decoding module. As a “plug-and-play” lightweight convolutional attention module, CBAM is composed of a Channel Attention Module (CAM) and a Spatial Attention Module (SAM) in series, as shown in the **Figure 5**.

In the channel attention module, both the operation of average-pooling and max-pooling are used simultaneously to generate average-pooled features and max-pooled features. Then, the two kinds of features are forwarded to a Multiple-layer Perceptron (MLP) to share feature. The output features of MLP are merged by element-wise summation and then activated by a sigmoid function to generate the channel attention feature maps *M_c_* (*F*). In short, the detailed operation to obtain channel attention can be computed as:


(2) $$M_{c}\left(F\right)=\sigma \left(MLP\left(AvgPool\left(F\right)\right)+MLP\left(MaxPool\left(F\right)\right)\right)$$

In the spatial attention module, the output features of CAM are taken as the input feature of SAM. Firstly, twice operations of pooling based on channels are used to aggregate channel information and generate average-pooled features and max-pooled features. Then, these features are concatenated and convolved by a standard convolution layer and produce the spatial attention feature map *M_s_* (*F*). In short, the detailed operation to obtain spatial attention can be computed as:


(3) $$M_{c}\left(F\right)=\sigma \left(f^{7\times 7}\left[AvgPool\left(F\right);MaxPool\left(F\right)\right]\right)$$

Where *σ* is an activation function of sigmoid, *F* denotes the input feature, and *f* ^7x7^ denotes a convolution operation with the filter size of 7 x 7.

In addition, some simple but effective adjustments are applied to the network improvement. Specifically: (a) The channels of input layer of the encoder were modified to ten layers because of the multispectral remote-sensing images containing more feature information than the traditional 3-channel RGB images (Zhao et al., 2022). (b) It is worth noting that the continuous large-scale upsampling is not conducive to obtain a satisfactory segmentation result, so we replaced the second 4-fold upsampling in thedecoder section with a 2-fold upsampling and a transpose convolution (Luo et al., 2021).

To extract richer multi-level features from the encoder, Deep-agriNet defined the low-level, mid-level and deep-level features in different scales to represent the extracted features by the Conv1, Maxpooling and Stage3 in the ShuffleNet v2, respectively. The specific process of this network could be described as below. Firstly, our network took 10-bands remote sensing images of 512 x 512 pixels as input and then processed by a 3 x 3 convolution layer with a stride of 2x 2 in the encoder module to obtain the low-level features of 256 x 256 pixels. These low-level features are then passed through a max-pooling layer to obtain mid-level features of 128 x 128 pixels. With the forward transmission of data, these intermediate features are down-sampled by multiple Shuffle blocks to obtain the deep-level features of 32 x 32 pixels. To obtain multi-scale fusion features, the deep-level features flow to an ASPP module of the decoder, where the input features are processed in parallel and concatenated by dilated convolutions with different dilation rates to capture the multi-scale information. Subsequently, the ASPP output information is passed through a single quadrupling up-sampling layer and a CBAM module in turn, resulting in 128*times*128 pixels feature with channel and spatial attention. These features are concatenated with the mid-level features in the encoder to reduce the loss of detail caused by multiple convolutions. After a 2-fold up-sampling, the concatenated features arerestored to 256 x 256 pixels and then concatenated with the low-level features in the encoder to fuse different level features from low to deep. Finally, the fused results are processed by transposed convolution to obtain the predicted image of pixel-level classification with 512 x 512 pixels.

### 2.4 Data acquisition

In this study, the remote sensing images of the T49RFQ area were cropped according to the size of 512 x 512 pixels, and 380 small-size patches were obtained in total. Since some patches did not contain crops or the cropped area is extremely tiny, we filtered and removed the data where the crop coverage was less than 30% of the total area. Finally, 100 patches were retained as dataset DS1. The same treatment was used on the T50SNA region to build dataset DS2, aiming to verify whether large-scale cropping affects the identification performance of the network. Then, we used the ArcMap program to annotate each pixel of the patches. When a pixel belongs to winter canola, its value is assigned as 1; when a pixel belongs to winter wheat, its value is assigned as 2, and in the rest cases, its value is assigned as 0. Finally, we obtained the same number of single-channel images corresponding to the patches on the dataset and used it as the annotation of the dataset. Prior to training, the dataset was split into training set and validation set randomly with a 7:3 ratio, which could reduce the imbalance of data and improve the network’s generalization ability.

### 2.5 Model training

During the training process, Back Propagation (BP) algorithm (LeCun et al., 2015) and Adaptive Moment Estimation (Adam) (Kingma and Ba, 2014) algorithm were adopted to speed up and optimize the convergence rate of Deep-agriNet. Since Deep-agriNet is a multi-class crop identification network, the multi-class cross-entropy loss function was used to calculate the loss between the predicted result and the true value of each epoch:


(4) $$Loss\left(y,\hat{y}\right)=-\sum_{i=1}^{N}y_{i}\log\;\hat{y_{i}}$$

where *y_i_* is the true value of a category whose value is 0 or 1, ŷ*_i_* is the predicted probability of the category whose value is distributed between 0 and 1,and *N* represents the category contained by the sample. Usually, the Learning Rate decays gradually during training, so we adopted the Polynomial Learning Rate Policy (Mishra and Sarawadekar, 2019) to dynamically adjust the learning rate:


(5) $$lr=base\_lr\times {\left(1-\frac{epoch}{max\_epoch}\right)}^{power}$$

where *lr* is the dynamic learning rate, *base_lr* is the baseline learning rate, *epoch* is the current number of iterations, *max_epoch* is the maximum number of iterations, and *power* is the power of the polynomial.

To prevent overfitting during training, we employed the operations of rotation, mirroring, and adding noise to augment the dataset to improve the generalization ability and robustness of the model. Meanwhile, the same operations were applied to the annotation as well.

### 2.6 Evaluation metrics

To evaluate the performance of Deep-agriNet, overall accuracy (OA), mean intersection over union (mIoU), and recall as evaluation metrics were used in this experiment. mIoU is one of the most basic metrics to evaluate the performance of semantic segmentation, and it represents the average of the ratio of the intersection and union of the predicted and true values for all classes:


(6) $$mIoU=\frac{1}{N}\sum_{i=1}^{N}\frac{P_{i}\cap G_{i}}{P_{i}\cup G_{i}}$$

where *N* is all categories of the sample including background. *P* and *G* are predicted and true pixels of a sample, respectively. OA represents the proportion of correctly classified pixels to all sample pixels. Recall represents the proportion of correctly classified pixels to all positive sample pixels:


(7) $$OA=\frac{TP+TN}{TP+TN+FP+FN}$$

(8) $$recall=\frac{TP}{TP+FN}$$

where *TP* is True Positive, indicating correct classification of pixels and positive predicted outcomes, *FP* is False Positive, meaning that the negative pixel is divided into positive samples, *TN* is True Negative, indicating the real background area is identified as the background area, and *FN* is False Negative, which represents the positive pixel is divided into negative samples.

Additionally, we introduced some metrics to evaluate the lightness of the network. The parameter is a commonly used evaluation metric for lightness of a network, which can measure the complexity of a model and the consumption of memory in computation. Theformula of parameter is shown as follow:


(9) $$parameter=K\times K\times C_{in}\times C_{out}$$

where the *K* x *K* means the size of kernel, and the *C_in_* and *C_out_* represents the number of input channels and output channels, respectively. In addition, the FLOPs which stands for floating-point of operations is a measure of network complexity. The FLOPs can be computed as:


(10) $$FLOPs=K\times K\times C_{in}\times H_{out}\times W_{out}\times C_{out}$$

where *H_out_* and *W_out_* represents the height and width of the output feature map. In this paper, we used the giga floating-point operations (GFLOPs, 10^9^ x *FLOPs*) to measure the complexity of network.

### 2.7 Hyperparameters and environment setting

To obtain more effective hyperparameters, we set the base learning rate to 0.0005, 0.001, 0.005, and 0.01. and batch size to 4, 8, 16, and 32, respectively. After several training sessions, the best results were obtained when the base learning rate was 0.001 and the batch size was 4. This experiment was trained on the Linux platform, and the deep learning framework used was Google’s open-source TensorFlow, and the GPU used for training was 24GB Nvidia GeForce GTX3090Ti.

## 3 Results

### 3.1 The comparison of lightweight between Deep-agriNet and other methods

To verify the effectiveness and superiority of the Deep-agriNet in terms of lightness, we calculated the parameter, GFLOPs and Inference Time(IT) for Deep-agriNet and other methods, and the results are shown in **Table 1**. From this table, it can be seen that HRNetv2 has the most parameters at 65.94M, while U-Net has the most GFLOPs at 450.64. In comparison, Deep-agriNet has significant advantages in evaluation metrics, parameters and GFLOPs, which are only 3.89M and 47.5. Moreover, the IT of Deep-agriNet is roughly comparable to that of U-Net (2.4 vs 1.8s).

**Table 1:** The lightweight metrics for Deep-agriNet and other methods.

Method	Backbone	Parameters(M)	GFLOPs	IT(s)
U-Net	VGG-16	24.9	450.64	**1.8**
PSPNet	ResNet-50	46.77	116.5	2.7
DeepLab v3+	Xception	54.2	103.16	3.1
HRNetv2	HRNetv2-W48	65.94	169.94	9.4
Deep-agriNet	ShuffleNetv2	**3.89**	**47.5**	2.4

The bold values indicate the highest scores in the experiments.

**Figure 6:**
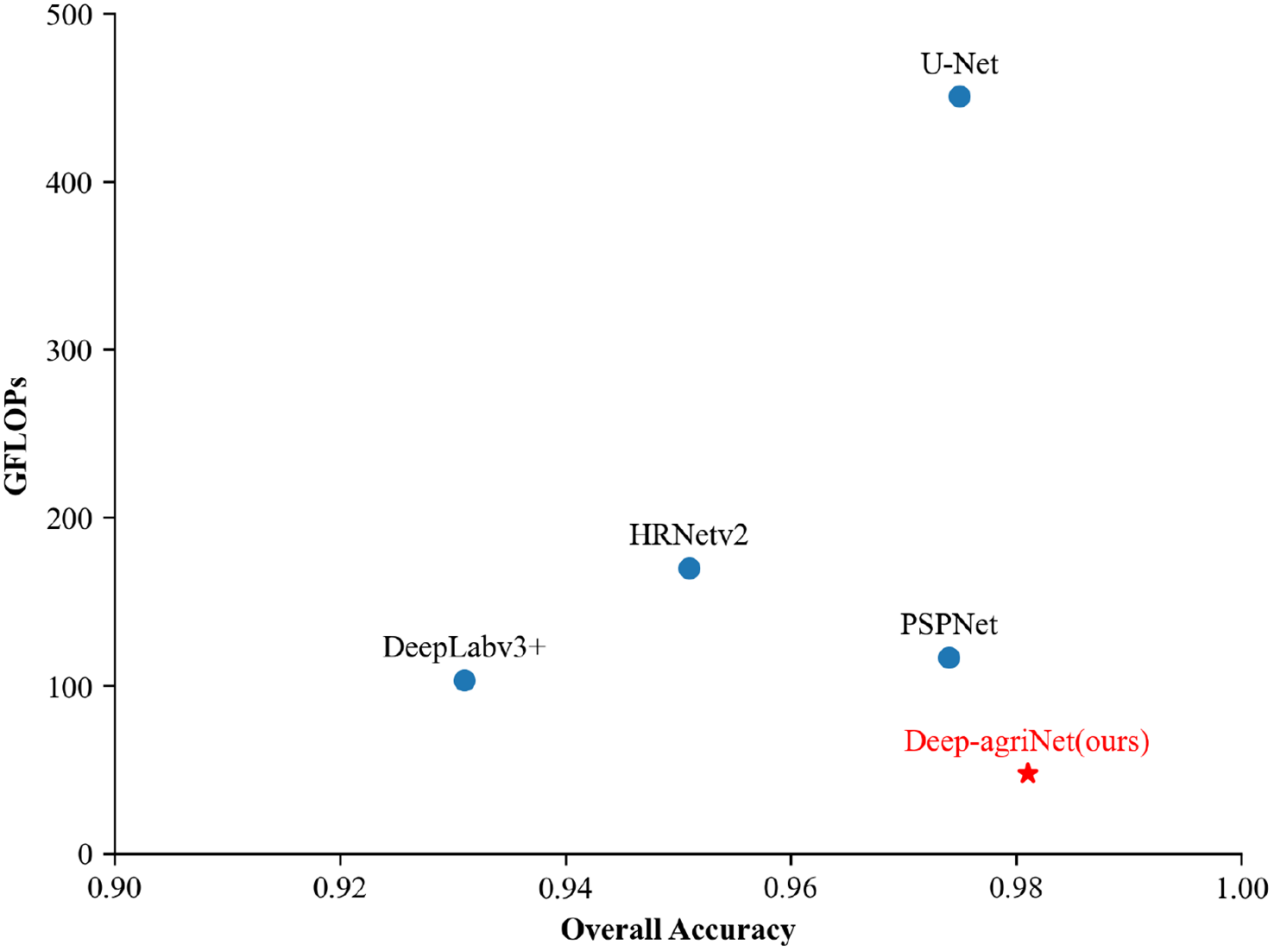
The scatter plot of accuracy and complexity of the networks on DS1.

In addition, we adopted a scatter plot better visualize the trade-off between accuracy and complexity and clarify the superiority of the proposed model Deep-agriNet. In the scatter plot, the x-axis indicates OA and the y-axis indicates GFLOPs. As shown in **Figure 6**, Deep-agriNet expresses the higher accuracy and lower GFLOPs than the other benchmark methods which means more lightweight and accurate of the proposed network.

### 3.2 The performance of Deep-agriNet for crop identification

**Figure 7:**
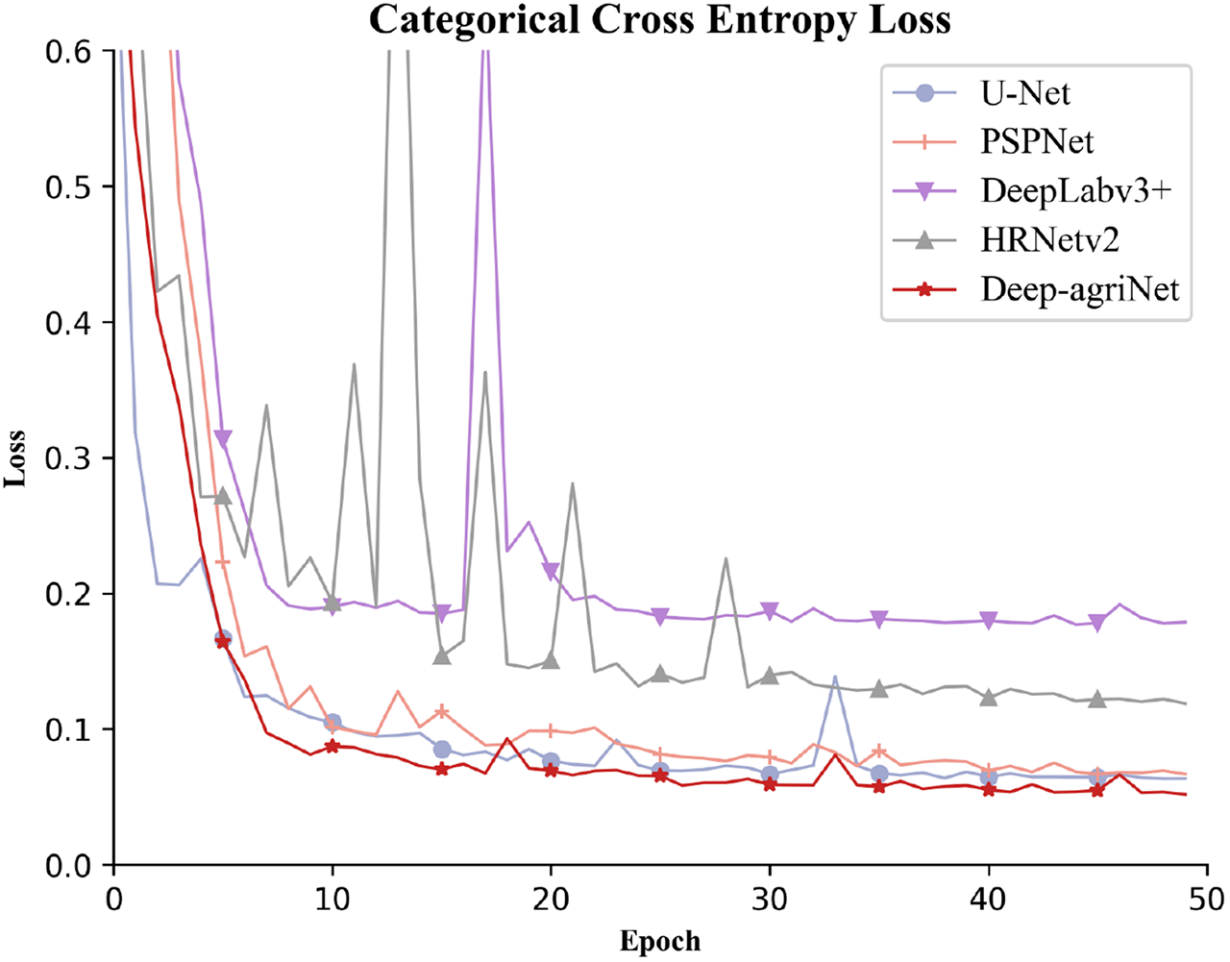
The convergence curves of Deep-agriNet and other methods.

The loss function curve can reflect the robustness and accuracy of the network. The smoother the curve is, the better the robustness of the model, and the smaller the loss value, the higher the accuracy of the model. The annotated DS1 was fed into Deep-agriNet and other methods for training. After 50 epochs, the loss convergence curves are shown in **Figure 7**. It clearly demonstrates that the cross-entropy loss tends to decrease with increasing epochs. According to the results in **Figure 7**, it can be seen that the Deep-agriNet based on DeepLab v3+ has more stable performance and higher accuracy in the training process.

To further analyze the performance of Deep-agriNet, this experiment compared these methods in terms of more evaluation metrics on accuracy, and the specific experimental results are shown in **Table 2**. From this table, it can be seen that Deep-agriNetperforms best in all aspects, where mIoU, OA, and recall is 0.972, 0.981, and 0.980. This results are significant improvement of 7.0%, 5.0% and 5.7% over the original DeepLab v3+, and slightly better than the next best performer U-Net by 0.8%, 0.6%,and 0.6%.

**Table 2:** Comparison of the identification results of different methods on DS1.

Method	mIoU	OA	Recall
U-Net	0.964	0.975	0.974
PSPNet	0.962	0.974	0.973
DeepLab v3+	0.902	0.931	0.923
HRNetv2	0.930	0.951	0.949
Deep-agriNet	**0.972**	**0.981**	**0.980**

The bold values indicate the highest scores in the experiments.

**Figure 8:**
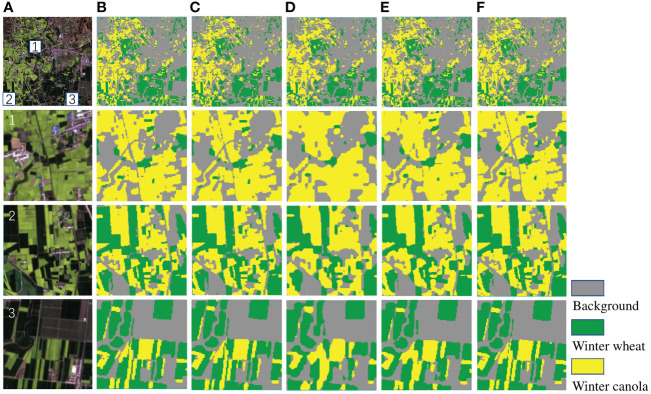
The original images and clipped regions of the experimental area on DS1 and the prediction results of different methods. **(A)** original image, **(B)** prediction result of U-Net, **(C)** prediction result of PSPNet, **(D)** prediction result of DeepLab v3+, **(E)** prediction result of HRNetv2, **(F)** prediction result of Deep-agriNet.

The **Figure 8** shows the identification results of Deep-agriNet and other methods for winter wheat and winter canola on DS1, where the yellow markers represent the winter canola planting area, the green markers represent the winter wheat planting area, and the gray markers represent the background. To show the prediction results of different methods more clearly, the marked patch1, patch2 and patch3 in the figure are enlarged to observe the details of the images. Comparing the original images and theprediction of multiple methods, the crop planting areas identified by U-Net, PSPNet and Deep-agriNet are highly consistent with the original images. Both the paths in the fields and the edges of the plots can be predicted with clarity. In contrast, the identification results of DeepLab v3+ and HRNetv2 are much worse. In patch1, the majority of the roads are not identified, and in patch2 and patch3, almost all the plots have different degrees of missing boundaries.

**Figure 9:**
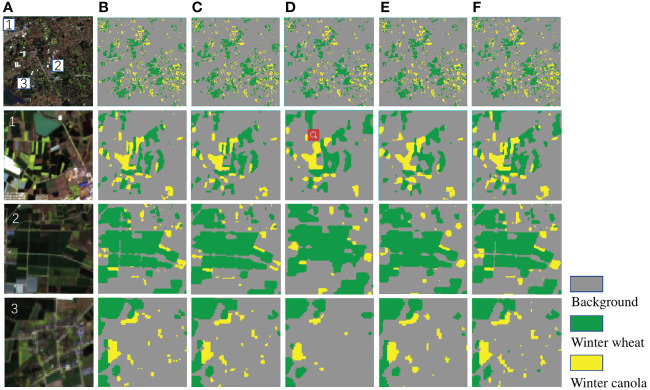
The original images and clipped regions of the experimental area on DS2 and the prediction results of different methods. **(A)** original image, **(B)** prediction result of U-Net, **(C)** prediction result of PSPNet, **(D)** prediction result of DeepLab v3+, **(E)** prediction result of HRNetv2, **(F)** prediction result of Deep-agriNet.

To further demonstrate the predictive capability of Deep-agriNet on the area with irregular small-scale plots, we trained and validated it on DS2. As shown in **Table 3**, Deep-agriNet still hold the best results in the respect of mIoU, OA, and recall with 0.961, 0.974, and 0.973, respectively. **Figure 9** shows the prediction results of different methods for winter wheat and winter canola on DS2. From the local magnification results of patch1, patch2 and patch3, Deep-agriNet still shows excellent performance on background identification and can predict the roads in the plots clearly. However, compared with the results on DS1, it can be obviously found that the model is less effective in irregular plots prediction and there is a slight phenomenon of boundaries missing. Comparing the identification results in **Figures 8**, **9**, Deep-agriNet performs better for crop identification with different planting scales.

**Table 3:** Comparison of the identification results of different methods on DS2.

Method	mIoU	OA	Recall
U-Net	0.956	0.967	0.963
PSPNet	0.954	0.965	0.965
DeepLab v3+	0.907	0.935	0.929
HRNetv2	0.921	0.945	0.943
Deep-agriNet	**0.961**	**0.974**	**0.973**

The bold values indicate the highest scores in the experiments.

### 3.3 Ablation study

**Figure 10:**
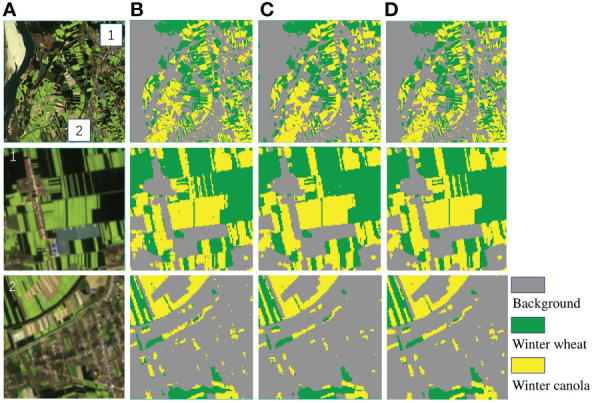
The original images and clipped regions of the experimental area on DS1 and the prediction results of the network with CBAM. **(A)** original image, **(B)** labeled image, **(C)** Deep-agriNet without CBAM, **(D)** Deep-agriNet (with CBAM).

To validate the role of CBAM in this network, The Deep-agriNet without CBAM was used as the baseline, and the two networks were trained with the same hyperparameters such as baseline learning rate and training epochs. As shown in **Table 4**, the network with CBAM is slightly improved in all aspects, including 0.8%, 0.6% and 0.6% for mIoU, OA and recall respectively on DS1. In addition, the parameters of the network only increased by 0.2M after adding CBAM. **Figure 10** shows the identification results of winter wheat and winter canola on DS1 before and after adding CBAM. As shown in the figure, CBAM was able to focus attention on the areas where winter wheat and winter canola were mixed and clearly identified plots of several pixel widths. In contrast, the network without CBAM was only able to identify fuzzy outlines but was unable to identify cross-planted plots.

**Table 4:** Comparison of the identification results of Deep-agriNet before and after adding CBAM.

Method	mIoU	OA	Recall	Parameters
Deep-agriNet without CBAM	0.964	0.975	0.974	3.87
Deep-agriNet	0.972	0.981	0.980	3.89

## 4 Discussion

### 4.1 Effects comparison between DeepLab v3+ and improved network

In this study, DeepLab v3+ was used as the base crop identification network, and a series of improvements were made on its basis. Finally, Deep-agriNet, the improved network, was applied to spring crop identification. Firstly, the backbone of DeepLab v3+, Xception, was replaced with ShuffleNet v2, a more advanced feature extractor. Based on this improvement, the identification accuracy was significantly improved, with mIoU, OA and recall improving by 6.3%, 4.5% and 5.1% on DS1. Meanwhile, the number of parameters and GFLOPs were also greatly optimized, much smaller than DeepLab v3+ and other methods. These performance improvements are mainly attributed to the following two factors: (a) the channel split method proposed by ShuffleNet v2 makes the input channels to be divided into two, with one part being passed down directly and the other part participating in the convolution operation, and finally the two parts are reassembled to reuse features. (b) ShuffleNet v2 transforms the elementwise add operation in depthwise convolution into a concatenation operation and replaces the grouped convolution with the ordinary convolution to greatly reduce the amount of computation. Then, A CBAM module was added between the encoder module and the decoder module in this study. Based on this improvement, the performance of mIoU, OA and recall has been improved slightly at the cost of a small computational cost. Specifically, on DS1, mIoU, OA, and recall are improved by 0.7%, 0.5%, and 0.6%, respectively, and the number of parameters hardly increased. As shown in **Figure 10**, although the improvement of evaluation metrics is quite small, the identification performance is improved considerably, and the phenomenon of missing edges and misidentified mixed-species regions is significantly improved compared with that before the improvement.

### 4.2 Result analysis of different areas

To investigate whether large-scale planting will affect the network performance, two regions, T49RFQ and T50SNA, were selected for this experiment, and Deep-agriNet was used to train DS1 and DS2 corresponding to the two regions. As shown in the **Tables 2**, **3**, Deep-agriNet has a better identification effect on the T49RFQ region of large-scale planting. Compared with the training results of DS2, the mIoU, OA and recall of DS1 increased by 0.9%, 0.7% and 0.7%, respectively. The author believes that the attention mechanism can capture the context dependence, and the data in DS1 has stronger spatial continuity. Even after multiple feature extraction, there is still a strong context dependence, which is beneficial to the decoder to infer the category of surrounding pixels through this dependence, and therefore the identification effect is improved.

## 5 Conclusions

In this paper, we proposed an improved lightweight network based on DeepLab v3+ and apply the network to spring crop identification. An advanced feature extractor, ShuffleNet v2, was used in this network to replace the backbone of DeepLab v3+. In addition, a CBAM combining channel and spatial attention mechanisms was added at the end of the encoder. In the decoder part of the original network, a 4-fold upsampling was modified to two adjacent 2-fold upsampling. To verify the performance of Deep-agriNet, two datasets with different planting scales were constructed for experiments. The experimental results show that the Deep-agriNet exhibits better performance on both datasets, and the parameters of the Deep-agriNet are only one-fourteenth of the original network. The Deep-agriNet can be applied not only for spring crop identification but also extended to other agricultural projects, such as crop yield prediction or crop disaster detection. However, to achieve this goal, further research on related work is needed to improve the algorithm so that the quantification of crop acreage can be achieved. In future work, we will try to use more advanced networks and larger agricultural datasets to meet more kinds of crop precision identification needs, and strive toserve our research results more effectively in the agricultural field.

## Data availability statement

The original contributions presented in the study are included in the article/supplementary material. Further inquiries can be directed to the corresponding author.

## Author contributions

YH, AM, LZ, and TX conceived the idea and designed the network. AM, LZ, ZJ, and YH contributed to collecting the dataset. YH and AM wrote the code, validated the method, and wrote the paper. TX, ZJ and YW revised the paper. All authors contributed to the article and approved the submitted version.
